# Association Mapping and Development of Marker-Assisted Selection Tools for the Resistance to White Pine Blister Rust in the Alberta Limber Pine Populations

**DOI:** 10.3389/fpls.2020.557672

**Published:** 2020-09-15

**Authors:** Jun-Jun Liu, Richard A. Sniezko, Robert Sissons, Jodie Krakowski, Genoa Alger, Anna W. Schoettle, Holly Williams, Arezoo Zamany, Rachel A. Zitomer, Angelia Kegley

**Affiliations:** ^1^ Canadian Forest Service, Natural Resources Canada, Victoria, BC, Canada; ^2^ USDA Forest Service, Dorena Genetic Resource Center, Cottage Grove, OR, United States; ^3^ Parks Canada, Waterton Lakes National Park, Waterton Park, AB, Canada; ^4^ Alberta Agriculture and Forestry, Edmonton, AB, Canada; ^5^ USDA Forest Service, Rocky Mountain Research Station, Fort Collins, CO, United States

**Keywords:** association mapping, *Cronartium ribicola*, limber pine (*Pinus flexilis*), major gene resistance (MGR), marker-assisted selection (MAS), plant effector-triggered immunity (ETI), single nucleotide polymorphisms (SNPs), white pine blister rust (WPBR)

## Abstract

Since its introduction to North America in the early 1900s, white pine blister rust (WPBR) caused by the fungal pathogen *Cronartium ribicola* has resulted in substantial economic losses and ecological damage to native North American five-needle pine species. The high susceptibility and mortality of these species, including limber pine (*Pinus flexilis*), creates an urgent need for the development and deployment of resistant germplasm to support recovery of impacted populations. Extensive screening for genetic resistance to WPBR has been underway for decades in some species but has only started recently in limber pine using seed families collected from wild parental trees in the USA and Canada. This study was conducted to characterize Alberta limber pine seed families for WPBR resistance and to develop reliable molecular tools for marker-assisted selection (MAS). Open-pollinated seed families were evaluated for host reaction following controlled infection using *C. ribicola* basidiospores. Phenotypic segregation for presence/absence of stem symptoms was observed in four seed families. The segregation ratios of these families were consistent with expression of major gene resistance (MGR) controlled by a dominant R locus. Based on linkage disequilibrium (LD)-based association mapping used to detect single nucleotide polymorphism (SNP) markers associated with MGR against *C. ribicola*, MGR in these seed families appears to be controlled by *Cr4* or other R genes in very close proximity to *Cr4*. These associated SNPs were located in genes involved in multiple molecular mechanisms potentially underlying limber pine MGR to *C. ribicola*, including NBS-LRR genes for recognition of *C. ribicola* effectors, signaling components, and a large set of defense-responsive genes with potential functions in plant effector-triggered immunity (ETI). Interactions of associated loci were identified for MGR selection in trees with complex genetic backgrounds. SNPs with tight Cr4-linkage were further converted to TaqMan assays to confirm their effectiveness as MAS tools. This work demonstrates the successful translation and deployment of molecular genetic knowledge into specific MAS tools that can be easily applied in a selection or breeding program to efficiently screen MGR against WPBR in Alberta limber pine populations.

## Introduction

Limber pine (*Pinus flexilis* James) is a native five-needle pine in western North America, naturally distributed from British Columbia and Alberta in Canada to southern California in the USA. Despite limited economic value, limber pine is a keystone species for high elevation montane ecosystems. Because of the species’ high tolerance to drought, high winds, and exposure, individual trees can live for over 1,000 years in harsh environments where few other conifers are distributed. In these environments limber pine provides essential ecosystem services, including slope stabilization, headwater streamflow control, and subalpine tree island formation. They provide cover that allows less exposure-hardy plants to establish and grow and provide both shelter and a nutritious food source for wildlife such as birds, bears, and small mammals (Schoettle, 2004; Tomback and Achuff, 2010). They are highly valued by recreationalists for their unique windswept beauty (Government of Alberta, 2014) and, as one of the world’s oldest living species, limber pine is also useful for dendrochronological studies (Millar et al., 2007).

Despite having a large geographic range, limber pine’s status is precarious in portions of its distribution. It is threatened by invasion of the lethal non-native fungal pathogen *Cronartium ribicola* (J.C. Fisch), outbreaks of native mountain pine beetle (*Dendroctonus ponderosae* Hopkins), and habitat loss resulting from changes to fire regimes and climate change. *C. ribicola* causes white pine blister rust (WPBR) in five-needle pines all around the world. Because of its high susceptibility to *C. ribicola*, in environments suitable for the pathogen, over 60 percent of limber pine trees in Alberta were observed to be infected during 2009 surveys in Canada, and WPBR caused over 40 percent mortality in many regions (Smith et al., 2013). The Government of Alberta and the Committee on the Status of Endangered Wildlife in Canada have designated limber pine as an endangered species (COSEWIC, 2014; Government of Alberta, 2014).

A species recovery plan has been prepared and executed for limber pine conservation and restoration in Alberta (Alberta Whitebark and Limber Pine Recovery Team, 2014). Genetic resistance is considered essential to several key strategies for WPBR management and restoration of native five-needle pines in North America, and great progress has been made in conservation and tree improvement programs for several species since programs began in the 1960s (Sniezko et al., 2008; Sniezko et al., 2014; Sniezko et al., 2020). Over the past few years, limber pine seed families have been collected and assessed for resistance-related phenotypic traits in controlled seedling inoculation trials. Major gene resistance (MGR) and quantitative disease resistance (QDR) to WPBR have been identified in populations not yet invaded by WPBR as well as those with high pre-selection from natural infection. MGR to *C. ribicola* was first identified in limber pine seed families originating from southern Wyoming to southern Colorado, USA, and its genetic locus was designated as *Cr4* (Schoettle et al., 2014). Recently, MGR was also independently found in a limber pine parent from Alberta (Sniezko et al., 2016), in stands 400 km south of the northern limit of the species. Because the two regions where MGR has been documented in limber pine are more than 1,100 km apart geographically, it is important for selection and breeding programs to know whether the MGR found in the USA and Canada is controlled by the same R locus (*Cr4*).

Traditional breeding approaches are effective in selecting and testing for genetic resistance to WPBR, but they can take decades to identify sufficient numbers of resistant parent trees and reliably produce large cone crops. In addition, it can be difficult using conventional methods for tree breeders to capture and transfer genetic variability of a suite of complex traits together, such as host resistance to pathogens and pests and other related adaptive traits. Genetic dissection of complex phenotypic traits is thus still an obstacle in forest genetics. In recent years, genomic resources have been developed by next generation sequencing approaches in a few five-needle pine species, including genome-wide marker discovery (Liu et al., 2014; Syring et al., 2016), high-density genetic maps (Jermstad et al., 2011; Friedline et al., 2015; Liu et al., 2019), transcriptome profiles (Lorenz et al., 2012; Liu et al., 2013; Gonzalez-Ibeas et al., 2016; Liu et al., 2016; Baker et al., 2018), and whole genome sequences (Stevens et al., 2016). These genomics resources and tools open a completely new avenue for the capture and utilization of genome-wide variability in breeding programs (Falk et al., 2018; Weiss et al., 2020).

Genotyping a subset of in-silico SNPs anchored *Cr4* on the *Pinus* consensus linkage group 8 (LG8) (Liu et al., 2016). Genes with positive selection implied in disease resistance or drought tolerance were identified in limber pine and three other five-needle pines as potential targets for breeding and selection of these traits (Baker et al., 2018). Fine-scale genetic mapping revealed orthologous R loci co-positioned with members of the nucleotide-binding site leucine-rich repeat (NBS-LRR) gene family that reflected evolutionary pressure (Liu et al., 2019). These genomic studies facilitated further characterization and practical utilization of genetic diversity in breeding genetic resistance to WPBR in five-needle pines. However, the genomic information available so far is still too limited to generate a sufficient foundation of knowledge required for genetic improvement of limber pine and other non-model forest conifers (Liu et al., 2016).

Here, we report an association study using unrelated open-pollinated limber pine seed families originating from Alberta to determine whether families expressing phenotypic signs of MGR have genotypic MGR markers. We also determine whether putatively resistant genotypes co-locate with previously identified *Cr4* known to occur in limber pine or whether they co-locate with other R genes. We aim to develop specific SNP markers that can be used for operational assays for MGR selection in breeding programs of limber pine to increase the frequency of WPBR-resistant genotypes.

## Materials and Methods

### Plant Materials and Resistance Assessment

Ten Alberta open-pollinated seed families (**Supplementary Table S1**) were sown at Dorena Genetic Resource Center (DGRC) in spring of 2016, including one family (PB #2) previously reported with MGR segregation (Sniezko et al., 2016) and nine families with unknown resistance levels. Needle samples were collected from each seedling and stored at −20°C before DNA extraction.

Of the five spore stages of the *C. ribicola* life cycle, only basidiospores are able to infect needles of five-needle pine species. They develop on the underside of *Ribes* spp. leaves (pathogen’s alternate host) during cool, wet weather conditions in late summer or early fall and infect the needles of five-needle pines. *Ribes* spp. leaves infected with a heterogenous mixture of *C. ribicola* genotypes were collected from multiple field locations in Oregon in the fall of 2016 and 2017. Since no virulent pathotype capable of overcoming limber pine MGR has yet been detected (Kinloch and Dupper, 2002), the *C. ribicola* sources used in these inoculations were assumed to be wild avirulent races.

Six and 18-month-old seedlings were inoculated in the fall of 2016 and 2017, respectively, using standard Dorena Genetic Resource Center protocols (Schoettle et al., 2014; Sniezko et al., 2016). Inoculation with *C. ribicola* basidiospores was performed in an inoculation room under controlled conditions at 18°C and 100% relative humidity. Study design was a randomized complete block, with 4 and 6 blocks for the 2016 and 2017 trials, respectively. Seedlings were transferred into the inoculation room two days prior to inoculation to acclimate to temperature and humidity conditions optimal for infection. Infected *Ribes* leaves were then placed on wire screens above the seedlings for the basidiospores to drop. Basidiospore shedding was monitored by checking microscope slides placed amongst the inoculation blocks. *Ribes* leaves were removed when basidiospore density reached a specific level (6000 or 3000 spores/cm^2^ in the 2016 and 2017 trials, respectively). Basidiospore germination averaged 99% for the trials. Seedlings remained in the inoculation chamber for about 48 h to promote spore germination and needle infection. After removal from the inoculation room, seedlings from the 2016 inoculation were then transferred to a greenhouse for the duration of the trial, while the seedlings from the 2017 inoculation were subsequently planted outdoors in boxes.

Disease development in the 2016 trial was assessed a total of 7 times during the period from October 2016 to December 2017. Each seedling was evaluated for disease symptoms on needles and stems including presence/absence of needle spots (lesions), spot types and number, as well as necrotic or abscising needles, presence/absence of cankers, aecia, and spermatia. Resistant or susceptible phenotypes were determined based on stem canker development as described previously (Schoettle et al., 2014; Sniezko et al., 2016). For the seedlings inoculated in 2017, number of needle spots was assessed approximately 8 months after inoculation, and presence/absence of spots, and number, type, and severity of stem infections as well as vigor was assessed approximately one year following inoculation.

Consistency for MGR phenotypic expression was estimated using χ^2^ tests by comparing phenotypic segregation of the same families inoculated in the two different years or with that from a previous report (Sniezko et al., 2016).

### Linkage Group (LG) Wide Association Study of Resistance to *C. ribicola* in Alberta Seed Families

Genomic DNA was extracted from needle tissues using a QIAGEN DNeasy Plant Mini kit. Previous studies mapped *Cr4* in USA sources on the *Pinus* consensus LG8, which positioned 25 and 775 genes, respectively (Liu et al., 2016; Liu et al., 2019). Here the limber pine LG8 in USA sources (**Supplementary Table S2**) was updated by map integration using MergeMap software (Wu et al., 2011). To address the question of whether the MGR observed in the Alberta seed families was different from *Cr4* found in the Colorado and Wyoming populations, genes were selected throughout LG8 for SNP genotyping. Based on SNPs within their coding regions, Sequenom iPlex genotyping arrays were designed and used to genotype seedlings of seed families identified with MGR segregation. SNP genotyping was performed using the Sequenom iPlex MassARRAY platform (Sequenom, San Diego, CA, USA; Gabriel et al., 2009) at the Genome Quebec Innovation Centre, McGill University, Montreal, Quebec.

The extent of linkage disequilibrium (LD) was investigated along LG8, and a matrix of the squared allele frequency correlations (*r^2^*) was constructed between all possible pairs of SNP loci with minor allele frequency (MAF) >5%. Association of markers with the resistant phenotype in the Alberta seed families was performed using a general linear model (GLM) and a mixed linear model (MLM) with the TASSEL software version 5.0 (Bradbury et al., 2007). In the GLM, *p*-values for marker effects were adjusted by 10^5^ permutations (Churchill and Doerge, 1994). To avoid spurious marker-trait associations that might result from population stratification or relatedness (Yu et al., 2006; Eu-ahsunthornwattana et al., 2014), the MLM included both fixed and random effect covariates which accounted for population structure and relatedness in genome-wide association studies (GWAS). In the MLM, proportions of individuals belonging to subpopulations were estimated as the Q matrix; average relationship between individual seedlings was estimated by kinship (K) calculated from genotypes of the SNP loci. Manhattan plots and quantile-quantile (Q-Q) plots were used to graphically present the results of association tests. The R squared values of markers (*r^2^*) were calculated and used to explain the proportion of phenotypic variation explained by each SNP locus. Associated SNPs and polymorphic genes were annotated based on their homologies to the available databases (NCBI-nr, PIR, KEGG, and GO) using BLAST2GO (Götz et al., 2008).

### Genetic Interaction Modeling

Gene-gene interactions were detected by the generalized MDR (GMDR) approach using the GMDR software package (Winham and Motsinger-Reif, 2011; Hou et al., 2019) to study the genetic interaction effect on the limber pine MGR. A 10-fold cross-validation procedure and 10-time random seed number were used to minimize potential for false positives. The best model was selected through an exhaustive search based on maximizing cross-validation consistency, training balance accuracy, and testing balance accuracy at a significance *p* < 0.05.

### Development of TaqMan Assays as MAS Tools

Based on results of the association analysis performed above, the top SNP loci with strong phenotypic association and potential R gene functional candidates were selected for design of TaqMan assays (**Table S3**). SNP genotyping was performed as described previously (Liu et al., 2020). TaqMan assays were first verified using genomic DNA extracted from megagametophyte tissues of at least one of the USA seed families previously used for genetic map construction (Liu et al., 2016), and then tested using the Alberta seedlings, including both the MGR-segregating and susceptible families identified in the present study. The genotype of each seedling was analyzed using the genotyping assays according to the manufacturer’s instructions (Applied Biosystems). A 7500 Fast Real-Time PCR system (Applied Biosystems) was used to run quantitative PCR. PCR hot-start was activated at 94°C for 15 min, followed by 40 cycles at 94°C for 20 s and 60°C for 60 s. Genotypes at each SNP locus were called using the TaqMan Genotyper Software (Applied Biosystems). To assess the potential application of TaqMan assays as MAS tools, their accuracy for phenotypic selection was calculated as the ratio of the number of individual samples with agreement between genotypes and phenotype to the number of all individuals.

## Results

### Phenotypic Segregation of Resistance Trait

**Figure 1:**
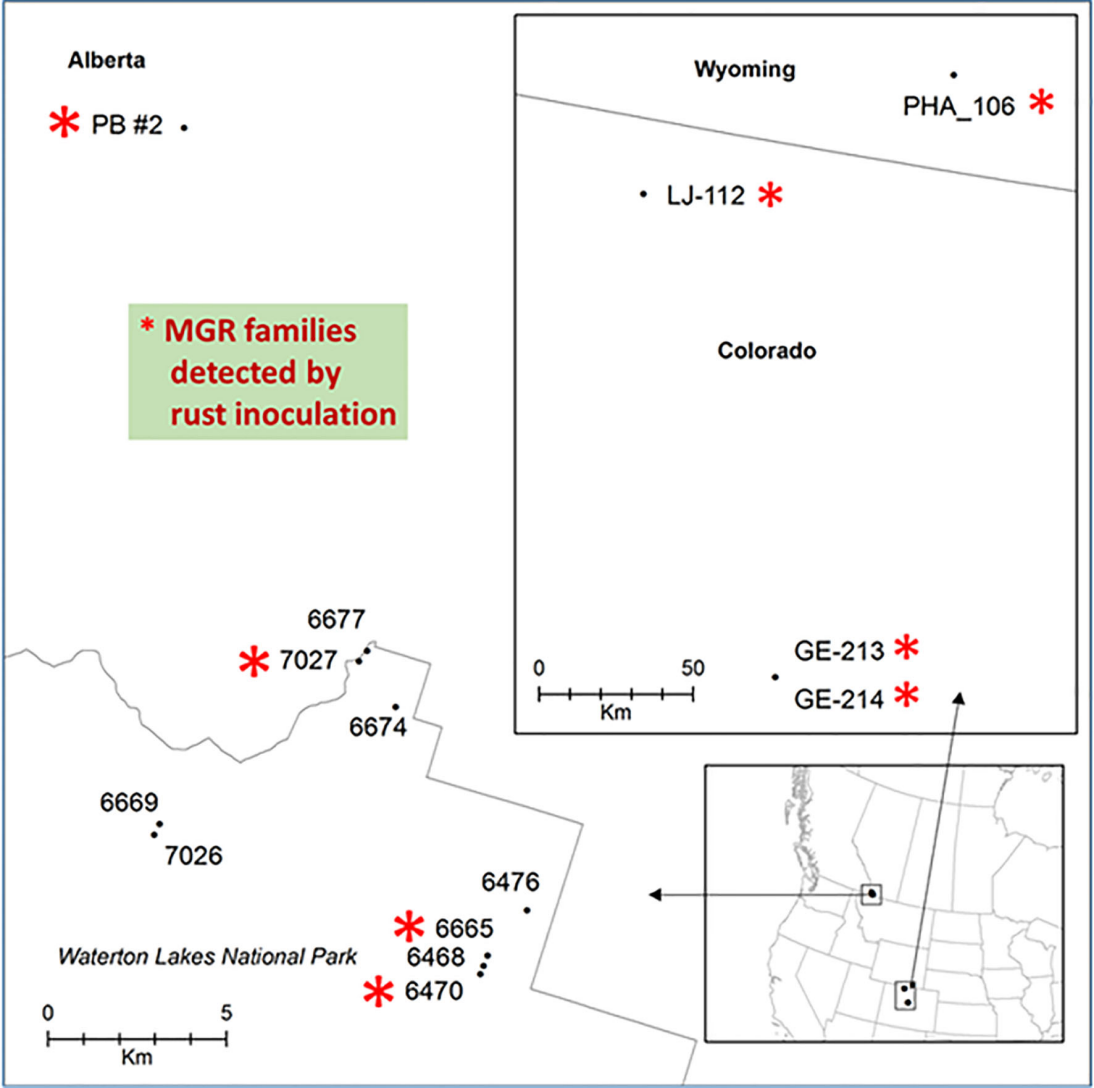
Geographic locations of limber pine seed families used in this study. Families with major gene resistance from Alberta (this study and Sniezko et al., 2016) and a subset of those found from Wyoming and Colorado (Schoettle et al., 2014) that were used in this study are labeled with red stars.

Phenotypic segregation for cankered and stem symptom-free seedlings was observed in four seed families: PB #2, #7027, #6470, and #6665 (**Figure 1**, **Table S1**). These families fit with the expected Mendelian segregation ratios at either 1:1 for families #7027, #6470, and #6665 (χ^2^ tested *p* values ranging from 0.68 to 1) or 3:1 for the family PB #2 (χ^2^ tested *p* = 0.13) (**Supplementary Table S1**), indicating that those seed trees are putatively heterozygous for MGR. In contrast, 95% to 100% of the seedlings from the other six seed families were cankered, and they were identified as susceptible (**Figure S1**, **Table S1**). These tested seed families were selected from fields where the wild stand infection rates were in a range of 83%~95% (**Table S1**). Identification of multiple MGR families from disease-free trees in the field by our controlled trials indicates that natural selection tends to increase frequency of resistance alleles in the survivors.

In a comparison of the 2016 and 2017 inoculation trials with greenhouse and outdoor conditions respectively, segregation ratios were highly consistent for families #7027, #6470, and #6665 (χ^2^ tested p values ranging from 0.29~0.62). PB #2 was not included in the 2017 inoculation trial, but its phenotypic segregation ratio from a 2014 inoculation (Sniezko et al., 2016) was also not significantly different from that of the 2016 trial (χ^2^ tested *p* = 0.80). This consistency of MGR phenotypic segregation indicates that there was no obvious environmental interaction for MGR phenotypic expression across Alberta MGR seed families.

### Linkage Disequilibrium (LD) of SNPs Along LG

**Figure 2:**
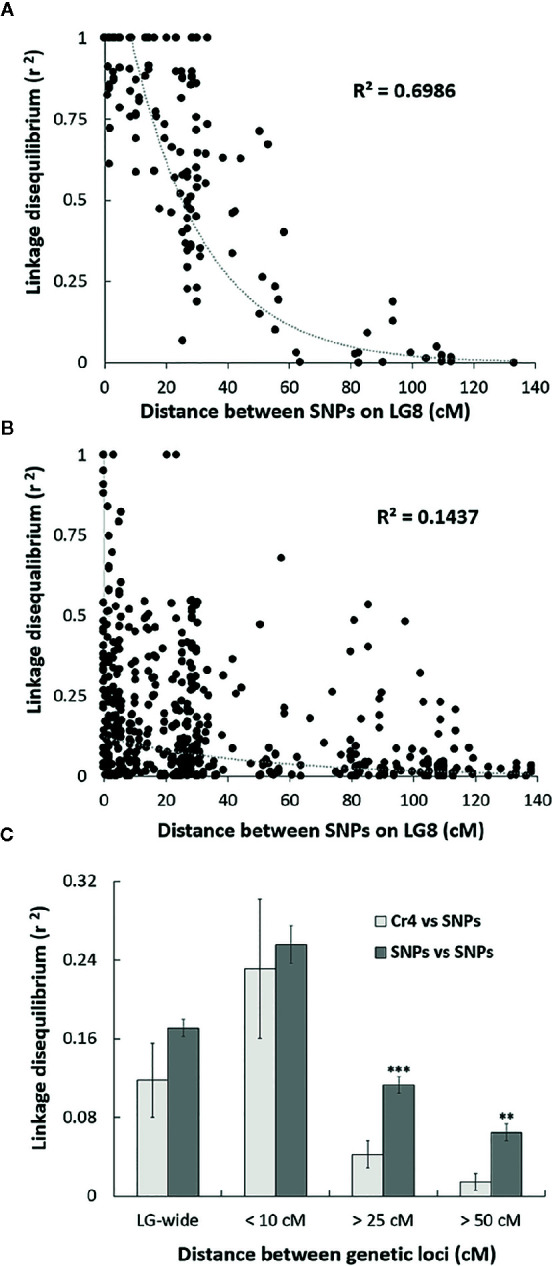
Scatter plot of the estimates (*r^2^*) of linkage disequilibrium (LD) decay for pairs of single-nucleotide polymorphisms (SNPs) in Alberta limber pine seed families with major gene resistance (MGR), with genetic distance in centimorgans (cM) within the Pinus consensus linkage group 8 (LG8). The curves are nonlinear regressions of LD decay (*r^2^*) onto distance in cM. **(A)** LD decay detected in the progeny of family PB #2; **(B)** LD decay detected in the progeny of all four MGR Alberta seed families; **(C)** Comparison of the LD decay estimates (*r^2^*) for pairs of SNPs and for pairs of *Cr4* coupling with one SNP locus. Pooled data from all surveyed SNPs were used to estimate LD decay based on distances between two loci at 10, 25, 50 cM, or LG-wide. Two and three stars indicate significant difference by t-test at *p < *0.01, and *p < *0.001, respectively.

Sequenom iPlex arrays were used for SNP genotype analysis across the four MGR-segregating seed families identified above. To reveal genetic correlations of SNP loci along LG, we first assessed LD levels between SNP loci using the *r^2^* statistic. As expected, high LD, or complete LD (*r^2^* = 1) was detected for the SNP pairs in the same genes or in different genes located at the same genetic position on LG8. Average *r^2^* between two SNP loci separated by distances less than 10 cM was 0.96 in the family PB #2 and 0.25 across the four families. The high average *r^2^* values at less than 10 cM indicate that the association mapping was powerful enough to detect causal loci at a relatively long distance in these limber pine populations using multiple seed families. A scatterplot of LD decay over genetic distance (cM) was generated to estimate the LD decay trend. Nonlinear regression revealed that LD decay was rapid as distances between the two markers increased (**Figure 2**), indicating that SNP markers with short-range LD likely have high reliability for MGR selection within limber pine seed families. When the distance between two SNP loci reached 25 cM, *r^2^* values decreased to 0.50 or 0.07 in the family PB #2 (**Figure 2A**) and across the four families (**Figure 2B**), respectively. Because a high mutation rate with frequent recombination events generally decreases LD, we further checked the *Cr4*-related LD across the four seed families and found that LD decay between the *Cr4* locus and SNP loci was faster than that between pairs of all SNP loci along the LG8, with the average *r^2^* value significantly lower (0.04 vs. 0.11, t-test *P* < 0.001) when the distance was over 25 cM (**Figure 2C**).

### LG-Wide Association Study Identifies *Cr4* as the R Locus in Four Alberta Seed Families

**Figure 3:**
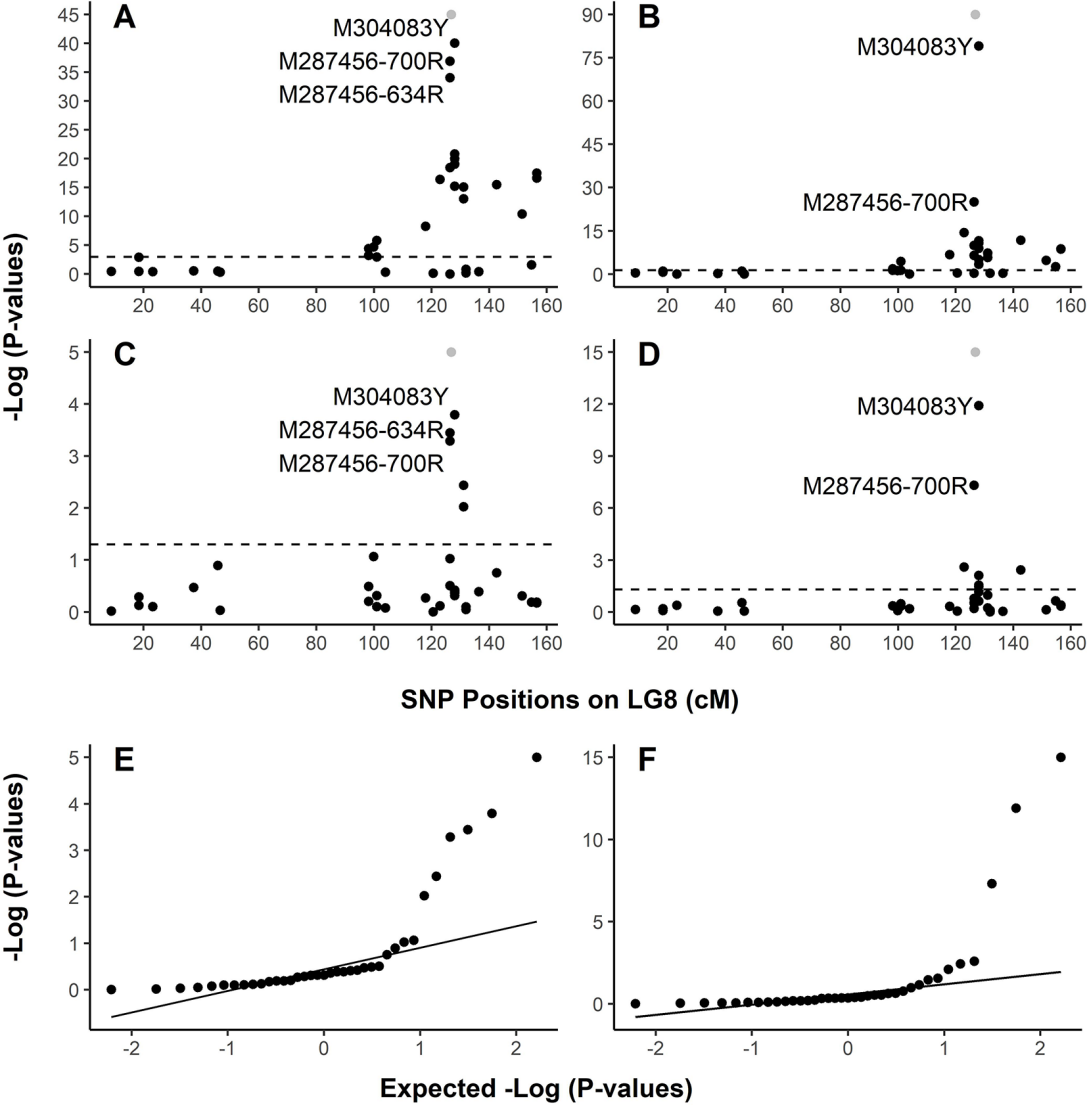
Graphical representation of test results showing strong association of SNPs with resistance to *Cronartium ribicola*. The Manhattan plots for resistance show –log10 p-values from linkage group 8 (LG8)-wide scan in Alberta seed families plotted against positions around the putative *Cr4* locus. The x-axis represents SNP locations (cM) on LG8, and the y-axis represents −log10 p-values from genotypic associations with the resistance phenotype. The dash lines mark the threshold of p-values in Manhattan plotting. **(A)** Association test using GLM with family PB #2; **(B)** Association test using GLM with all samples of four Alberta seed families; **(C)** Association test using MLM with family PB #2; **(D)** Association test using MLM with all samples of four Alberta seed families. Quantile-quantile (Q-Q) plots showing the enrichment of association signals with observed p-values against the expected distribution under the null hypothesis. In the Manhattan plots **(A**–**D)**, Cr4 was included in grey dots to show its position on LG8. LG8 was updated by integrating genetic maps described previously (Liu et al., 2016; Liu et al., 2019). **(E)** Q-Q plot showing results of MLM run using family PB #2. **(F)** Q-Q plot showing results of MLM run using all samples of four Alberta seed families.

In family PB #2, LG-wide association analysis using the general linear model (GLM) detected a total of 23 SNP loci (**Table S4**) in significant association with the resistant phenotype. *r^2^* for these 23 SNP markers ranged from 0.099 to 0.839 (*p* < 0.05 after correction by 1 × 10^5^ permutations). The resistance-associated SNPs were distributed in 17 genes localized on LG8 from positions 18.33 cM to 156.48 cM. Based on the LG8 genetic map updated in the present study (**Figure S1**, **Table S2**), M287456 was on one side and M304083 was on the other side of *Cr4* at 0.43 and 1.11 cM, respectively. The Manhattan plots of –Log10 (*p* values) or marker-Rsq (*r^2^* values) on a genome scale with marker positions (cM) across LG8 (**Figure 3A**, **Figure S2**) each had a single peak of significant marker-trait associations, near 126.80 cM where *Cr4* and its candidate genes were localized for MGR to *C. ribicola*. SNP M304083Y displayed the strongest association with the resistant phenotype (*r^2^* = 0.839), followed by M287456-634R and M287456-700R with *r^2^* = 0.730 (**Figure S2**).

Association analysis across all four open-pollinated seed families detected 18 SNP loci within 13 genes in significant association with the resistant phenotypes (**Figure 3B**, **Figure S2B**, and **Table S5**). These GLM-based results were well supported by an association analysis using a mixed linear model (MLM) (**Figures 3C, D**). As presented by Manhattan plots and quantile-quantile plots, MLM-based association tests detected 5 and 7 SNP loci with significant association with MGR in the PB #2 family and all seedlings across the four segregating families, respectively (**Figures 3E, F** and **Supplementary Table S5**). All association tests consistently showed co-localization of spikes of association with *Cr4*, indicating that the R locus was the same (*Cr4*) or very closely positioned in Canadian and USA seed families despite the large geographic distance between them.

**Figure 4:**
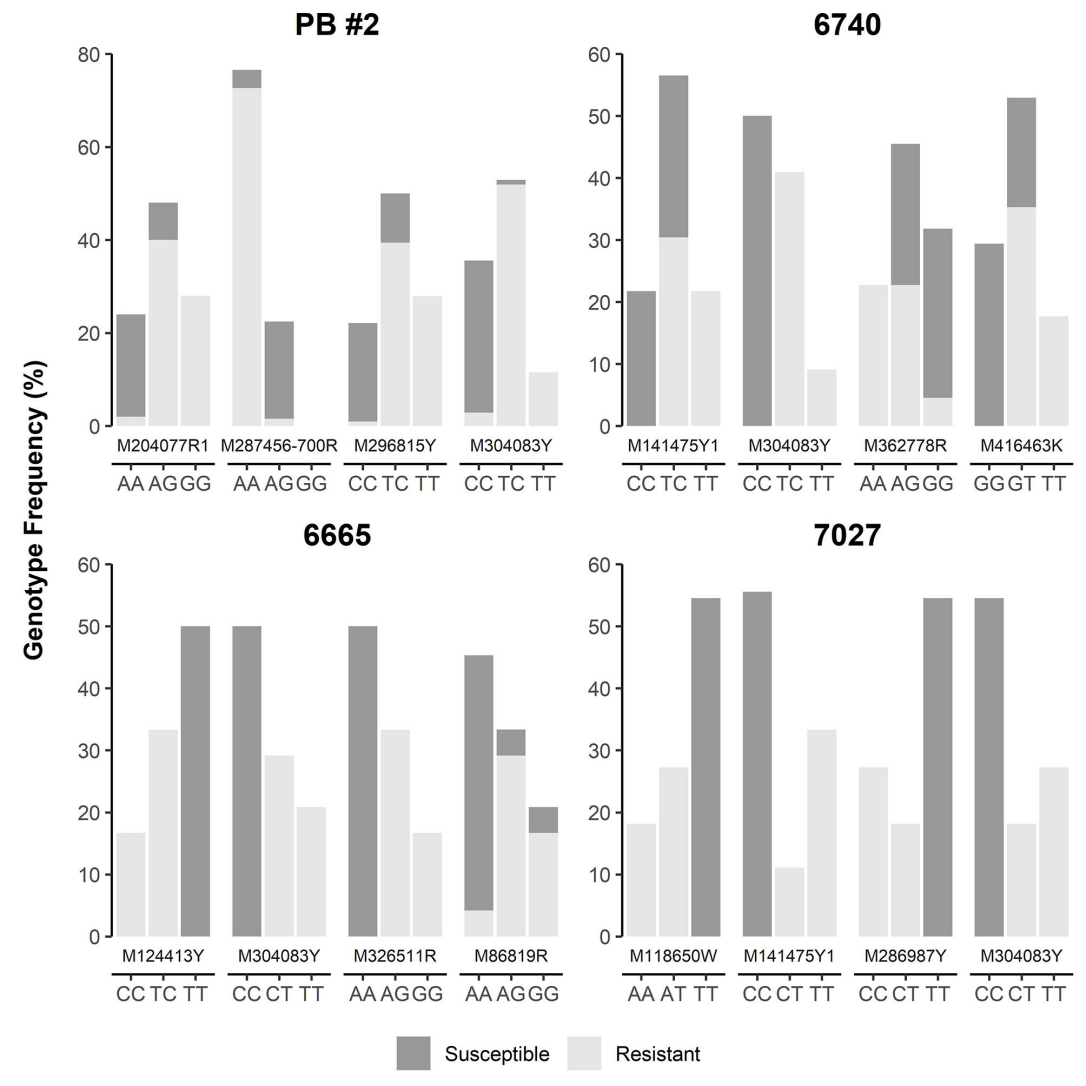
Genotype frequency of the SNP loci that showed co-segregation or significant association with resistance to *Cronartium ribicola* in four Alberta open-pollinated seed families.

SNP loci were further checked manually in each of four MGR-segregating open-pollinated seed families from Alberta, ranking a total of 12 SNPs, including M287456-700R and M304083Y as identified above by GLM and MLM, as the top SNPs with strong phenotypic association in at least one seed family (**Figure 4**). Most of them were positioned within 5 cM of *Cr4* (**Supplementary Figure S1**). Three and four SNPs co-segregated with resistant phenotypes in families #6665 (M124413Y, M304083Y, M286917Y, and M326511R) and #7027 (M118650W, M141475Y1, and M304083Y), respectively. Of these SNPs, M124413Y and M326511R were not detected by GLM and MLM runs across all four seed families due to the small sample size (34 seedlings) of seed family #6665.

### Annotation of Polymorphic Genes Associated With MGR in Alberta Limber Pine Families

In total, 25 associated SNPs distributed across 19 unigenes were identified. Nine polymorphic genes were localized within 5-cM of *Cr4* (**Table S5**). All 19 associated unigenes showed significant homology hits in the Arabidopsis proteome by BLAST analysis (**Table S6**). Three genes had unknown function (M355530, M445775, and M124413), and 16 genes were assigned putative biological functions by BLAST-2-GO-based gene annotation. These included two R analogs of the NBS-LRR family (M463406 and M287456), two regulators for signal transduction pathways (M187937: RAB GTPase homolog G3D and M438219: little nuclei1-LINC1), three transcriptional factors (TFs; M296815: CCCH-type zinc finger protein with ARM repeat domain, M141475: GRAS family transcription factor, and M160798: Tesmin/TSO1-like CXC domain-containing protein). Five associated genes were involved in defense responses to biotic and abiotic stressors, including expansin-like B1 (M176778), galactinol synthase 1 (M204077), stress responsive α/β barrel domain protein (SRBP, M304083), pectin acetylesterase (PAE, M286987), and 6-phosphogluconate dehydrogenase (6PGDH, M259257). The remaining four genes were revealed to have functions involving ribosomal protein L12 (M444092), cytidine/deoxycytidylate deaminase (DCTD, M286987), nucleotide-diphospho-sugar transferase (M362778), and proline-rich spliceosome-associated (PSP) family protein (M118650).

### Gene–Gene Interactions Associated With Limber Pine MGR

Modeling interactions between MGR-associated genes revealed three gene–gene models with the highest order. Consistent with results from the GLM and MLM-based association tests (above), a one‐gene model was first identified with the SRBP gene (M304083Y) for MGR; it scored 10/10 for cross‐validation consistency. A two-gene interaction was identified between SRBP (M304083Y) and DCTD (M286987Y), while a three-gene interaction was identified among SRBP (M304083Y), NBS-LRR (M287456-700R), and 6PGDH (M259257Y) which scored 9/10 and 7/10 for cross‐validation consistency, respectively. The 10-fold cross validation for significance test showed all top tree models with *p* = 0.001 (**Table S7**).

GMDR analysis calculated interactive variables in the score distributions, with a positive score for the MGR resistant group and a negative score for the susceptible group (**Figure S3**). The one-gene SRBP model detected uncertainty of MGR phenotypes for trees carrying SRBP genotypes (M304083-TC and CC) (**Figure S3A**). In the two-gene interactions of SRBP x DCTC, four genotypes in combinations of SRBP (M304083-TT and TC) and DCTD (M286987-CC and TC) were detected as the resistance interactive variables in the GMDR score distributions while seedlings with genotype SRBP (M304083-TC) in combinations with DCTD genotypes M286987-TC showed a higher potential to be susceptible than to be resistant (**Figure S3B**), decreasing false-positive diagnoses from the one-gene model. The uncertainty of MGR phenotypes resulting from SRBP genotype M304083-TC and CC was minimized in the three-gene model of SRBP, NBS-LRR and 6PGDH interactions (**Figure 3C**). SRBP genotype M304083-TC in combination with NBS-LRR genotype M287456-700AG was modulated to be susceptible (−0.6 vs. 0.0) while SRBP genotype M304083-CC in combination with 6PGDH genotype M259257CC was modulated to be resistant (0.4 vs. 0.0).

### Development of TaqMan Arrays as MAS Tools for Breeding of Limber Pine Resistance

Because M304083Y had the highest marker-Rsq (*r^2^*) across all four families and M287456 was a NBS-LRR candidate, SNP loci within both genes were selected to design TaqMan assays (**Table S4**). SNP genotypes detected using TaqMan assays and the Sequenom MassARRAY iPLEX platform were consistent, indicating that both technologies were highly reliable for SNP genotyping in limber pine. The TaqMan assays revealed genotypic segregation at the M304083-485Y locus in the progeny populations of four seed families, showing that the parent trees of all four MGR families have a T/C genotype at the M304083-485Y locus. In their progenies, T/T and T/C were linked to the resistant phenotype while C/C was linked to the susceptible phenotype. The genotype-phenotype matching rates were 100% in the family #7027, 97.4% in the family PB #2, 97.1% in the family #6665, and 95.8% in the family #6470 (**Figure 4**), consistent with the results from the GLM and MLM-based association tests and GMDR modeling. Among parents of the six susceptible families (**Figure 1**, **Table S1**), five had the homozygous genotype C/C, and one had the heterozygous genotype C/T at the M304083-485Y locus. These results demonstrated that this TaqMan assay was an excellent MAS tool for MGR selection for the four Alberta MGR seed families. The TaqMan assay M287456-700R was valid with a genotype-phenotype matching rate of 95.4% in seed family PB #2, with A/A linked to the resistant phenotype and A/G linked to the susceptible phenotype. However, this locus was detected as a G/G genotype in the majority of progeny of the other three MGR seed families. Notably, the M304083 gene encoded a protein with unknown function and the gene M287456 was a *Cr4* candidate encoding a putative NBS-LRR protein (**Table S5**). Additional studies will be needed to confirm the functional contributions of these proteins to disease resistance in WPBR pathosystems.

## Discussion

### Association Mapping in Limber Pine Open-Pollinated Populations

The present study documented MGR in three additional Alberta seed families as well as the previously identified PB #2, identifying new resistant germplasm to incorporate into the limber pine restoration program (Alberta Whitebark and Limber Pine Recovery Team, 2014). An association approach was used to map MGR because these four seed families originated in different areas and were open-pollinated with unknown pollen sources from natural stands. Because the presence of LD or allelic association is a prerequisite for association mapping, LD decay among SNPs using several Alberta open-pollinated limber pine families was examined. LD decay was detected with *r* ^2^ = 0.5 over 25 cM in the seed family PB #2, a similar level to biparental populations in other species (Flint-Garcia et al., 2003). We consistently found that the extent of LD was much higher in one open-pollinated family than that in the combined progeny of multiple parent trees in limber pine, indicating the latter had a greater genetic variability. Despite this high diversity, LD decay in a progeny population across four limber pine families (~10 cM at *r^2^* > 0.1) was much slower than in diversity panels as reported in other conifers (Plomion et al., 2014; Westbrook et al., 2015).

Association study results are commonly affected by the diversity of sampled individuals and population size. While previous studies assessed populations with <100 samples (Hart and Griffiths, 2015; Chang et al., 2016; Corwin et al., 2016), we sampled over 200 seedlings across four seed families; this allowed successful detection of SNPs associated with Alberta limber pine MGR. GWAS uses LD to identify markers associated with traits of interest that are segregating in target populations. Although unrelated populations are typically used in GWAS with case control designs, family-based GWAS designs are widely used to determine association between markers and traits within families. Compared to GWAS with case control designs using unrelated populations, GWAS with family-based designs is more powerful for detecting associated markers by avoiding artifacts of stratification at the population level (Lange et al., 2008). We used family-based populations without cryptic population structure, eliminating false positive marker-trait associations (Lipka et al., 2015). We conducted association analysis using both GLM and MLM. The latter assessed familial relatedness among individuals using a kinship matrix for family-based association studies of quantitative or binary traits (Eu-ahsunthornwattana et al., 2014; Hart and Griffiths, 2015). The MLM identified fewer SNPs with significant associations than did GLM, suggesting that MLM may be overly conservative (Wang et al., 2012). Our association study confirmed that the MGR in seed families from both the USA and Canada was controlled either by the same R gene (*Cr4*), or different R gene alleles positioned very close to each other.

### Molecular Mechanisms Underlying Limber Pine Resistance *to C. ribicola*

Association of DNA variants with phenotypes facilitates elucidation of gene function and regulation as well as allelic architecture of complex traits (Neale and Savolainen, 2004). Based on the gene-for-gene model for plant disease resistance (Flor, 1971), we expected to identify limber pine R alleles that express host receptors interacting with effectors secreted by *C. ribicola* cells. Most well characterized R proteins in molecular plant-microbe interactions belong to the superfamilies of NBS‐LRR and receptor-like protein kinases (RLK), acting either as intracellular or cell-surface receptors for reorganization of pathogenic effectors (Glazebrook, 2005). Of the two NBS-LRR genes associated with limber pine MGR, M287456 was mapped within a genetic distance < 0.5 cM of *Cr4*. Two M287456 SNPs were among the most significantly associated with the MGR phenotype. However, M287456 showed only limited similarity to its closest homologs in the sugar pine (*P. lambertiana* Dougl.) genome (Stevens et al., 2016) and western white pine (*P. monticola* ex D. Don) transcriptome (Liu et al., 2019), suggesting that it might have evolved recently, after diversification of the subgenus *Strobus*. This data supports our hypothesis that M287456 is the top R candidate for MGR against WPBR in Alberta seed families.

NBS-LRR proteins specifically target pathogenic effector proteins, initiating effector-triggered immunity (ETI) through signal transduction networks (Chisholm et al., 2006). A series of immune signaling components and TFs contribute to ETI downstream responses, including ion flux, oxidative reactive oxygen species (ROS) burst, activation of defense-responsive genes that facilitate syntheses of pathogenesis-related proteins, callose deposition, and hypersensitive response (HR)-related programmed cell death, which is a phenotypic expression of a typical resistance trait (Buscaill and Rivas, 2014). We identified two signaling components (homologs of RAB GTPase and LINC1) in association with MGR in Alberta limber pine. Small GTPases are well characterized as signaling components in activating defense responses through regulation of reactive oxygen species (ROS) during plant-microbe interactions (Rivero et al., 2019). For example, GTPase RabA4c overexpression enhanced callose deposition at an early infection stage and caused complete penetration resistance to the virulent powdery mildew (Ellinger et al., 2014). Arabidopsis LINC1 is a positive PTI regulator and plays a key role in jasmonic acid signaling and glucosinolate biosynthesis; *linc1* mutants showed basal immunity towards *Pseudomonas syringae* and enhanced resistance to *Botrytis cinerea* infection (Jarad et al., 2020).

Among three MGR-associated TF genes in limber pine, the CCCH-type zinc finger proteins with ARM repeat domains constitute a TF family with multiple roles, including maintaining vegetative growth, enhancing stress tolerance, and stress-induced reproduction in response to several different stressors (Blanvillain et al., 2011). In western white pine, two CCCH-type zinc finger genes were upregulated in response to *C. ribicola* infection, including one exclusively responsive in the incompatible *Cr2-avcr2* interaction (Liu et al., 2013). In Arabidopsis, one family member (oxidative stress 2, OXS2) is required for salt tolerance by activating other downstream differentially expressed genes in the defense response (Jing et al., 2019). The GRAS TF family consists of multiple members that have different functions in plant development, signal transduction, and stress response. Several GRAS TFs are involved in regulation of cutin biosynthesis to promote mycorrhizal colonization (Xue et al., 2015). Induction of GRAS TF by fungal signals regulates the expression of downstream genes required for cell morphology changes during the host responses to obligate biotrophic fungi (Heck et al., 2016). Overall, the signaling and TF genes involved in limber pine MGR may imply the occurrence of cross-talk at different levels to integrate multiple signal pathways during white pine-blister rust (WP-BR) interactions.

Our study identified five associated genes involved in ETI downstream responses. Among them, a SNP of gene M304083, encoding a stress responsive α/β barrel domain protein, was most significantly associated with MGR, followed by four other genes with activities of expansin, galactinol synthase, PAE, and 6PGDH. The stress-response α/β barrel domain proteins have been known to be upregulated in response to salt stress (Gu et al., 2004; Zhan et al., 2019). The expansin superfamily, including expansin-like B, causes cell-wall loosening activity during cell expansion and other developmental events where cell-wall structures are modified (Sampedro and Cosgrove, 2005). The Arabidopsis expansin‐like A2 (EXLA2) gene was identified by its down‐regulation in response to infection by *Botrytis cinerea*, and its mutant showed enhanced resistance to necrotrophic fungi and hypersensitivity to abiotic stress (Abuqamar et al., 2013). Similarly, a western white pine expansin transcript was down-regulated in the early incompatible (*Cr2-avcr2*) WP-BR interactions (Liu et al., 2013). Galactinol synthase participates in the biosynthesis of oligosaccharides, promoting plant stress tolerance to heat, chilling, salinity and oxidative damage (Panikulangara et al., 2004; Nishizawa et al., 2008). In contrast to expansin, galactinol synthase was transcriptionally upregulated in the early incompatible (*Cr2-avcr2*) WP-BR interactions (Liu et al., 2013). PAEs catalyze the deacetylation of pectin, and decreased pectin acetylation results in increased resistance to microbial pathogens in transgenic plants (Pogorelko et al., 2013). As revealed by transcriptome analysis of mutants, Arabidopsis PAE9 activity affected transcript expression of 56 genes, mainly involved in oxidation-reduction reactions (Kloth et al., 2019). 6PGDH is the third enzyme of the pentose phosphate pathway and plays an essential role in oxidative stress defense (Hanau et al., 2013). 6PGDH was highly accumulated following pathogen infection in wheat, suggesting it is a key component in the activation of host defenses (Li et al., 2017). These reports provide further evidence to support the hypothesis that defense responsive genes play an important role in limber pine resistance. This large set of host genes works together to coordinate various molecular mechanisms underlying limber pine MGR. However, further studies are required to more fully characterize the related molecular mechanisms contributing to host resistance in WPBR pathosystems.

### Development of MAS Tools for Limber Pine Resistance Screening

Major gene resistance to WPBR is an excellent case study for SNP development and validation of MAS tools because it is a well-studied trait in five-needle pine breeding programs (Sniezko et al., 2014). Of 25 associated markers that were mapped on LG8 in at least one of the USA MGR seed families (Liu et al., 2016; Liu et al., 2019), the SNP marker M304083Y showed a 97.5% genotype-phenotype matching rate across all tested seedlings from four Alberta families, and it was successfully converted into a TaqMan assay. Our modeling of gene-gene interactions revealed a potential to increase the selection accuracy of MAS tools by combining multiple genetic loci. Based on sensitivity, precision, specificity, design flexibility, and cost (Tajadini et al., 2014; Broccanello et al., 2018), the TaqMan SNP genotyping technology was selected for use in limber pine and other conifers (Liu et al., 2017; Liu et al., 2020). As a stress responsive α/β barrel domain protein, M304083 may only function in the PTI downstream response in the WPBR pathosystem. This gene provides an excellent genomic target for selective breeding of resistant individuals in related seed families.

The TaqMan assay developed from a SNP site of the NBS-LRR gene M287456 was revealed to be valid for MGR selection in only one of the tested seed families (PB #2). Due to low level expression of the NBS-LRR gene, only a partial fragment of M287456 was assembled in the limber pine transcriptome. Our previous exome-seq analysis mapped over 600 NBS-LRR gene sequences in limber pine and most of them were mapped in clusters throughout the genome. However, few NBS-LRR genes were mapped in the *Cr4* region (Liu et al., 2019). The NBS-LRR family is highly polymorphic and usually under diversifying selection pressure during plant genome evolution (Zhang et al., 2016). We also found that *Cr4*-related LD decay was much faster than the decay for all other pairs of the detected SNPs at >25 cM, suggesting more frequent recombination at this *R* locus. Availability of M287456 allowed fine dissection of the *R* locus by sequencing and mapping of its homologs. Further work is needed to explore nucleotide variations throughout the full M287456 gene and its homologs. Additional SNP sites within this NBS-LRR *Cr4*-candidate and its duplicated paralogs would have high potential for development of new MAS tools through high throughput technologies, such as resistance gene enrichment sequencing (RenSeq) (Jupe et al., 2013). Coupled with QTL or GWAS studies for host resistance to WPBR, localizing all members of the NBS-LRR family across the genome would allow fine-scale genetic dissection of limber pine MGR and QDR to *C. ribicola*. As more novel genes are identified with strong phenotypic association, characterizing SNP loci within them for design of robust and deployable MAS tools will enhance limber pine improvement programs.

Selection of favorable alleles or allelic combinations is a lengthy process requiring breeding of outcrossing conifer species. In genotypic selection focused on a few genes with large phenotypic effects (such as MGR), application of MAS tools will enable shortened breeding cycles by allowing precise prediction of phenotypes for those genes. In limber pine breeding, ideal MAS tools will allow testing of needles of parent trees in the wild to determine if they have MGR, minimizing the need to wait to collect a viable seed crop, grow and inoculate seedlings, or at least reducing the number of seed families that must be tested to find MGR. Another important use of MAS tools for MGRs is for genotypic confirmation to support pyramiding of multiple resistance mechanisms. Some types of QDR may also exhibit stem symptom-free traits. Infection by virulent pathotypes may negate the MGR and allow observation of QDR among seedlings. Therefore, MAS tools may be applied to within-family selection for joint MGR and QDR as well as to forward selection in seed orchards when incorporating new parents that have both MGR and QDR genotypes.

MAS tools are too narrow for general use in more complex traits which are controlled by multiple genes. Although modeling of multiple gene interactions may expand the application of MAS tools to larger areas with more complex genetic backgrounds, genomic selection is a more attractive approach for breeders to select and predict complex traits with minor gene effects. Genetic characterization of genes or QTLs contributing to various complex traits of interest is an essential prerequisite for genomics-based breeding. GWAS and QTL identification for disease resistance are still relatively rare in five-needle pines. When SNPs underlying important quantitative traits are available from future GWAS and QTL mapping, their conversion into similar genotyping arrays will be useful for genomic selection in breeding programs. Our MAS tools and genotyping arrays, as well as previously reported genetic maps, offer valuable resources for future GWAS, QTL mapping, and genomic selection of these complex traits in limber pine and other related five-needle pines.

Future analysis aimed at assaying genetic variation across the whole genome is likely to facilitate a much more comprehensive elucidation of the genetic basis of phenotypic variation. Over 150 additional Alberta limber pine families from parents in heavily infected stands are currently under evaluation for WPBR resistance. As resistance related phenotypic data becomes available from conservation and breeding programs in the coming years, it would be interesting to expand the study to a wider geographical area where other resistance mechanisms may be present. Because the Sequenom-based SNP genotyping arrays developed here were verified for their application in association mapping of *Cr4*, these genotyping arrays can be applied to any limber pine families to search for additional R genes. Novel R genes will be confirmed in those families if all *Cr4*-linked SNPs of the genotyping arrays show no association with resistant phenotypes.

## Data Availability Statement

All relevant data is contained within the article. Gene sequence data for design of Sequenom iPLEX MassARRAY are shown in **Supplementary Material Table S4**.

## Author Contributions

J-JL, RAS, RS, and JK conceived the research. RS, GA, JK, and RAS provided the genetic materials. RAS, RZ, and AK conducted the disease ratings and analyzed phenotypic data. J-JL, HW, and AZ conducted SNP genotyping and analyzed genotypic data. AS shared SNP data and some genotypes of the limber pine species for the study. J-JL wrote the original draft with contributions from all authors. J-JL and RAS supervised the project. All authors contributed to the article and approved the submitted version.

## Funding

This research was supported in part by the CFS-Pest Risk Management Program and funding from Waterton Lakes National Park of Canada.

## Conflict of Interest

The authors declare that the research was conducted in the absence of any commercial or financial relationships that could be construed as a potential conflict of interest.

## Supplementary Material

Supplementary Figure 1 Fine-scale genetic linkage map of the limber pine *Cr4* locus. Genetic distances between two adjacent loci are labeled in centimorgans (cM).

Supplementary Figure 2 Scatter plot of the squared correlations of allele frequencies (*r ^2^*) versus distance in cM across Pinus consensus linkage group 8 (LG8). Each dot represents a SNP, with the X-axis showing their locations on LG8 and Y-axis showing the marker-Rsq values (*r ^2^*) squared for each SNP marker. The putative *Cr4* gene was included with an expected *r ^2^* value at 1. **(A)** Progeny of the seed family PB #2; **(B)** Progeny of all four Alberta seed families.

Supplementary Figure 3 Gene-Gene interactions for association with major gene resistance detected by GMDR analysis. In each cell, the left bar represents a positive score for the resistant group and the right bar a negative score for the susceptible group. Resistant cells were indicated by dark shading, susceptible cells by light shading. For SRBP (M304083Y), 0 represents TT, 1 represents TC, and 2 represents CC genotype. For NBS-LRR (M287456-700R), 0 represents AA, 1 represents AG, and 2 represents GG genotype. For both DCTD (M286987Y) and 6PGDH (M259257Y), 0 represents CC, 1 represents TC, and 2 represents TT genotype. **(A)** one-gene model with SRBP (M304083Y); **(B)** two-gene model with SRBP (M304083Y) and DCTD (M286987Y); **(C)** three-gene model with SRBP (M304083Y), 6PGDH (M259257Y), and NBS-LRR (M287456-700R).
