# Supplementary figures and images for: Association Mapping and Development of Marker-Assisted Selection Tools for the Resistance to White Pine Blister Rust in the Alberta Limber Pine Populations
Source: Front Plant Sci. 2020 Sep 15;11:557672. doi: 10.3389/fpls.2020.557672 (PMC7522202; doi:10.3389/fpls.2020.557672)

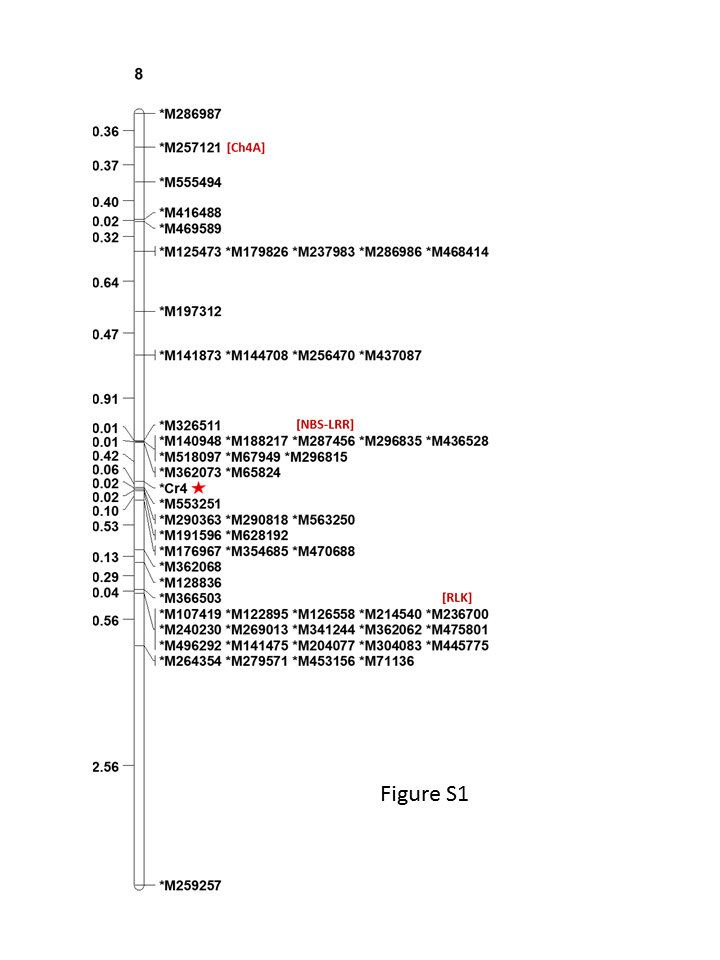

Supplement: Supplementary Figure 1 — Fine-scale genetic linkage map of the limber pine Cr4 locus. Genetic distances between two adjacent loci are labeled in centimorgans (cM). [file Image_1.tif]

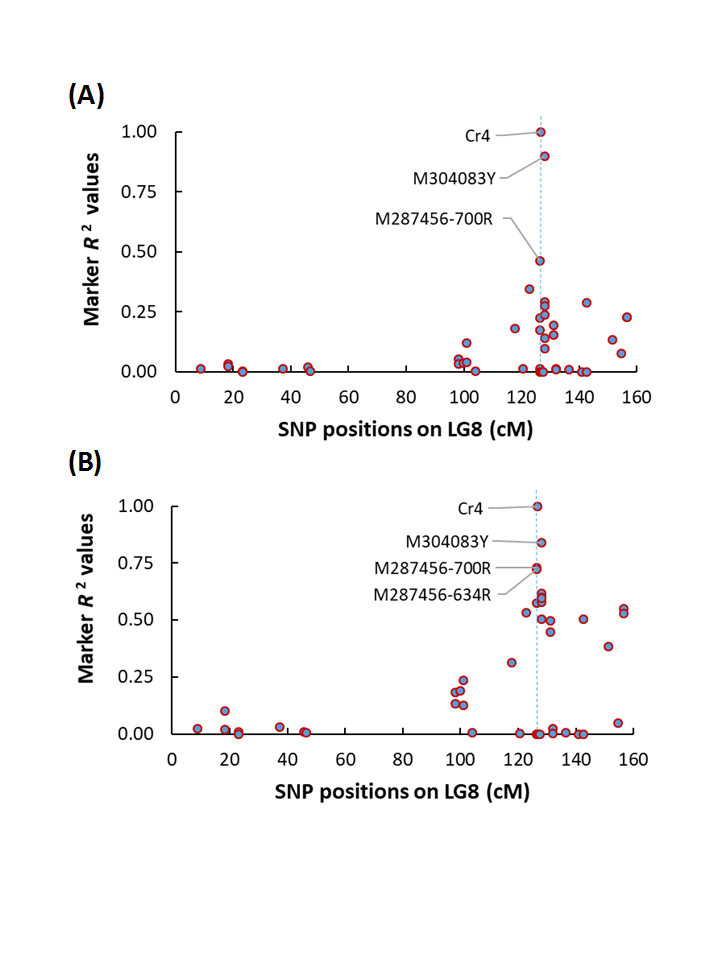

Supplement: Supplementary Figure 2 — Scatter plot of the squared correlations of allele frequencies (r 2) versus distance in cM across Pinus consensus linkage group 8 (LG8). Each dot represents a SNP, with the X-axis showing their locations on LG8 and Y-axis showing the marker-Rsq values (r 2) squared for each SNP marker. The putative Cr4 gene was included with an expected r 2 value at 1. (A) Progeny of the seed family PB #2; (B) Progeny of all four Alberta seed families. [file Image_2.tif]

## Slide 1
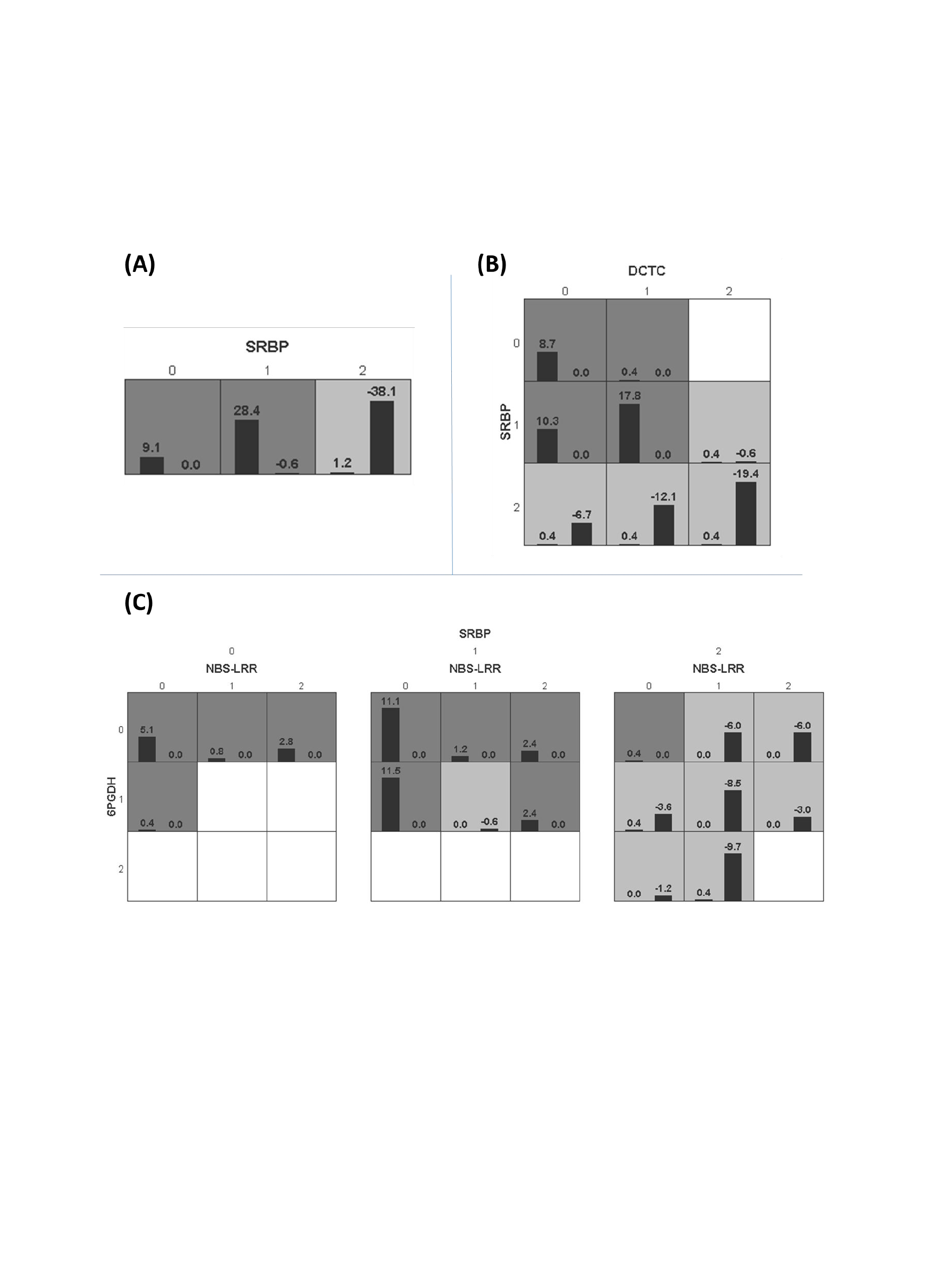

Supplement: Supplementary Figure 3 — Gene-Gene interactions for association with major gene resistance detected by GMDR analysis. In each cell, the left bar represents a positive score for the resistant group and the right bar a negative score for the susceptible group. Resistant cells were indicated by dark shading, susceptible cells by light shading. For SRBP (M304083Y), 0 represents TT, 1 represents TC, and 2 represents CC genotype. For NBS-LRR (M287456-700R), 0 represents AA, 1 represents AG, and 2 represents GG genotype. For both DCTD (M286987Y) and 6PGDH (M259257Y), 0 represents CC, 1 represents TC, and 2 represents TT genotype. (A) one-gene model with SRBP (M304083Y); (B) two-gene model with SRBP (M304083Y) and DCTD (M286987Y); (C) three-gene model with SRBP (M304083Y), 6PGDH (M259257Y), and NBS-LRR (M287456-700R). [file Presentation_1.pptx]
